# Protective Mechanism of Humanin Against Oxidative Stress in Aging-Related Cardiovascular Diseases

**DOI:** 10.3389/fendo.2021.683151

**Published:** 2021-06-10

**Authors:** He Cai, Yunxia Liu, Hongbo Men, Yang Zheng

**Affiliations:** The Cardiovascular Center, First Hospital of Jilin University, Changchun, China

**Keywords:** humanin, oxidative stress, aging-related cardiovascular diseases, redox signaling pathways, metabolic abnormalities

## Abstract

Physiological reactive oxygen species (ROS) are important regulators of intercellular signal transduction. Oxidative and antioxidation systems maintain a dynamic balance under physiological conditions. Increases in ROS levels destroy the dynamic balance, leading to oxidative stress damage. Oxidative stress is involved in the pathogenesis of aging-related cardiovascular diseases (ACVD), such as atherosclerosis, myocardial infarction, and heart failure, by contributing to apoptosis, hypertrophy, and fibrosis. Oxidative phosphorylation in mitochondria is the main source of ROS. Increasing evidence demonstrates the relationship between ACVD and humanin (HN), an endogenous peptide encoded by mitochondrial DNA. HN protects cardiomyocytes, endothelial cells, and fibroblasts from oxidative stress, highlighting its protective role in atherosclerosis, ischemia–reperfusion injury, and heart failure. Herein, we reviewed the signaling pathways associated with the HN effects on redox signals, including Kelch-like ECH-associated protein 1 (Keap1)/nuclear factor erythroid 2-related factor 2 (Nrf2), chaperone-mediated autophagy (CMA), c-jun NH2 terminal kinase (JNK)/p38 mitogen-activated protein kinase (p38 MAPK), adenosine monophosphate-activated protein kinase (AMPK), and phosphoinositide 3-kinase (PI3K)/protein kinase B (Akt)-Janus kinase 2 (JAK2)/signal transducer and activator of transcription 3 (STAT3). Furthermore, we discussed the relationship among HN, redox signaling pathways, and ACVD. Finally, we propose that HN may be a candidate drug for ACVD.

## Introduction

With the increase of an aging population, aging-related cardiovascular diseases (ACVDs) confer a heavy economic burden on society (1, 2). Oxidative stress is involved in the pathogenesis of ACVD (3–5), such as atherosclerosis (6), myocardial infarction (7), and heart failure (8), by contributing to apoptosis, hypertrophy, and fibrosis. Oxidative phosphorylation in mitochondria is the main source of reactive oxygen species (ROS). Increased ROS levels destroy the dynamic balance between oxidative and antioxidation systems, leading to oxidative stress damage. Thus, suppressing ROS generation is a potential strategy for the treatment of ACVD.

Humanin (HN), an endogenous active peptide encoded by mitochondrial DNA, has been shown to be related to ACVD (9) (1): the serum HN level negatively correlates with age (10, 11); (2) HN reduced H_2_O_2_-induced oxidative stress damage in myocardial cells and isolated myocardial mitochondria by promoting the expression of antioxidant defense system proteins (12) and inhibiting the activity of complexes I and III of the electron transport chain (13); (3) HN reduced ROS production, protecting endothelial cells from oxidative stress damage induced by abnormal glycolipid metabolism (14–16); (4) HN restored chaperone-mediated autophagy (CMA) by regulating heat shock protein 90 (Hsp90) and decreasing ROS production, thereby protecting cardiomyocytes and fibroblasts from oxidative stress damage (17); (5) HN upregulates the expression of antioxidant enzymes, preserving the heart function after myocardial infarction in an ischemia–reperfusion injury model by reducing myocardial cell death and the area of myocardial infarction (18–20). Of note, there are only a few studies that investigated the redox signaling pathways associated with HN. Moreover, there is no detailed review about the redox signaling pathways involving HN in ACVD. Herein, we reviewed the regulation of HN expression and the main downstream signaling pathways involved in oxidative stress and discussed the relationship among HN, the redox signaling pathways, and ACVD. Finally, we propose that HN may be a candidate drug for ACVD.

## The Origin and Functions of HN

Mitochondrial DNA encodes mitochondria-derived peptides (MDPs). MDPs include HN, mitochondrial ORF of the twelve S c (MOTS-c), and small humanin-like peptides (SHLPs) 1–6. HN was first discovered in patients with Alzheimer’s disease (AD). HN suppresses neuronal cell death, suggesting it may be a candidate drug for AD (21, 22). HN is transcribed from a 75-bp open reading frame sequence of the large 16S mitochondrial ribosomal RNA (rRNA) in the cytoplasm, generating a 24-amino-acid peptide with the sequence, Met-Ala-Pro-Arg-Gly-Phe-Ser-Cys-Leu-Leu-Leu-Leu-Thr-Ser-Glu-Ile-Asp-Leu-Pro-Val-Lys-Arg-Arg-Ala (22–24). However, *HN* mRNA is translated into a 21-amino-acid peptide in mitochondria, without the last three amino acid residues found in the HN translated in the cytoplasm. Notably, both variants contain basic amino acids in the N-terminal and C-terminal with similar functions (21, 25, 26). HN mediates a variety of intracellular and extracellular signaling pathways and plays multiple protection functions. It inhibits the translocation of proapoptotic proteins, such as Bax, Bid, and tBid, into mitochondria by binding to them. Furthermore, HN suppresses cytochrome C release and the formation of apoptotic bodies, thereby inhibiting mitochondria-dependent apoptosis (27, 28). The Golgi apparatus and endoplasmic reticulum are required for HN release (22), and released HN binds to two kinds of receptors on the cell membrane, the cell membrane trimer comprising CNTFR, WSX-1, and the 130-kDa gp130, and formyl peptide receptor-like 1 (FPRL1) (29, 30). After binding to the trimer receptor: (1) HN activates AMP-activated protein kinase (AMPK), further suppressing the mammalian target of rapamycin (mTOR) and nuclear factor kappa B (NF-*κ*B) signaling pathways; (2) HN activates the phosphoinositide 3-kinase (PI3K)/protein kinase B (AKT)-Janus kinase 2 (JAK2)/signal transducer and activator of transcription 3 (STAT3) signaling pathway; and (3) HN inhibits the c-jun NH2 terminal kinase (JNK)/p38 mitogen-activated protein kinase (MAPK) signaling pathways, protecting cellular and mitochondrial functions (31–37). Moreover, HN activates FPRL1 receptor and extracellular signal-regulated kinases (ERK1/2) (30). HN has many protective functions, such as anti-aging, inhibition of myocardial fibrosis, regulation of mitochondrial homeostasis, anti-inflammation, improving metabolism, regulation of the redox system, and autophagy promotion. It protects the retinal segment epithelium from oxidative stress-induced aging (38) and inhibits myocardial fibrosis in aged mice (39). HN promotes mitochondrial biogenesis and regulates mitochondrial homeostasis (40) and it decreases the expression of tumor necrosis factor alpha (TNF-α), interleukin (IL)-1β, and IL-6, to inhibit inflammation (41). Additionally, HN has potential in the treatment of diabetes by improving *β*-cell survival (32), promoting insulin secretion (42), and improving insulin resistance (10). HN also promotes chaperone-mediated autophagy (17), decreases ROS production, promotes the expression of antioxidant proteins and maintains the redox system balance (12).

## HN and Oxidative Stress

### Regulation of HN Expression Under Oxidative Stress

**Figure 1:**
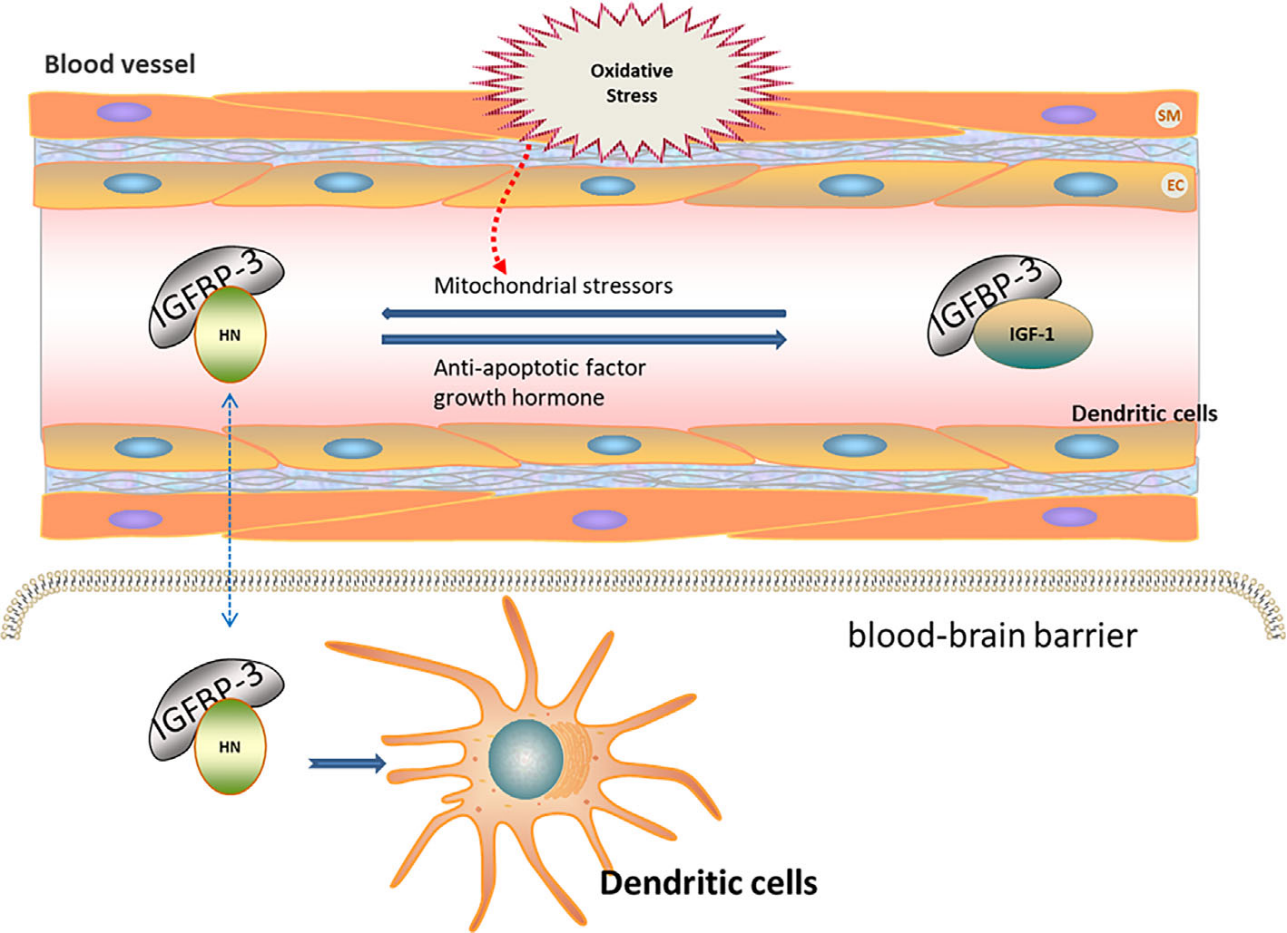
Regulation of HN expression under oxidative stress. HN levels in peripheral blood are regulated by IGF and IGFBP. IGFBP-3 is the main component of IGFBP in peripheral blood, with high affinity to HN. IGFBP-3 may transport HN through the blood–brain barrier (Blue dotted arrow), thereby reducing ROS and further protecting nerve cells. Growth hormone down-regulates HN levels in peripheral blood through the high expression of IGF-1. Mitochondrial stressors, such as serum deprivation and chemotherapeutic drugs, increase HN expressions. Anti-apoptotic factors decrease HN expressions. HN levels under oxidative stress may be regulated by increasing IGFBP-3 levels or inhibiting IGF-1 levels.

Oxidative stress contributes to ischemia–reperfusion injury (43). The expression of HN is increased after ischemia–reperfusion injury in mice, indicating the association of HN expression with oxidative stress (44). HN levels in the skeletal muscle and in the plasma of humans and mice negatively correlate with the increase in age (10, 45). HN levels in the peripheral blood are regulated by insulin-like growth factor (IGF) and IGF-binding protein (IGFBP). IGFBP-3 is the main component of IGFBP in the peripheral blood, with high affinity to HN (46, 47). The decrease in HN in the peripheral blood of AD patients suggests that HN in the peripheral blood may have protective effects on the nervous system (48). IGFBP-3 may transport HN through the blood–brain barrier, thereby reducing ROS production and further protecting nerve cells (49, 50). Growth hormone down-regulates HN levels in the peripheral blood through high expression of IGF-1 (51). Mitochondrial stressors, such as serum deprivation and chemotherapeutic drugs, can increase HN expression (52–54). In contrast, antiapoptotic factors decrease HN expression. Tripartite motif protein 11 (TRIM11) degrades intracellular HN through the proteasome pathway (49, 55). Therefore, HN levels under oxidative stress may be regulated by increasing IGFBP-3 levels or inhibiting IGF-1 levels (**Figure 1**).

### HN Regulates Redox Signaling Pathways

HN promotes the expression of antioxidant enzymes that inhibit ROS production through intracellular and/or extracellular pathways (56, 57). Intracellularly, (1) HN protects mitochondrial function by inhibiting electron transport chain complexes I and III and decreasing ROS formation; (2) HN activates the Kelch-like ECH-associated protein 1 (Keap1)/nuclear factor erythroid 2-related factor 2 (Nrf2) signaling pathway and the expression of antioxidant stress elements of nuclear genes through the reverse signal transduction between mitochondria and nuclei; (3) HN activates CMA through HSP90, promoting the absorption of oxidation products and further reducing ROS production. Extracellularly, HN binds to receptors on the cell membrane, triggering downstream signaling pathways, including the JNK/p38 MAPK, AMPK, and PI3K/AKT-JAK2/STAT3 signaling pathways, thereby promoting autophagy, reducing ROS production, and protecting the function of cells and mitochondria (**Table 1**). Understanding these signaling pathways is essential for understanding the antioxidant effect of HN in ACVD

**Table 1:** Regulation and biological effect of HN signaling pathways on oxidative stress.

Signaling pathway	Model	stimulate	Intervention	Direct effects	Indirect effects	Reference
**Humanin-Nrf2**	• Human ovarian cells	• PCOS	• HNG	Increase antioxidant enzyme Reduce ROS	Protect ovarian cells	(58)
**Humanin-CMA**	• HUVECs	• OX-LDL	• Gly-14 humanin	restores cathepsinD function promotes autophagic degradation	Inhibit lipid and cholesterol aggregation Aging cell protection	(59)
	H9C2 MN9D NIH3T3	Paraquat Rotenone H2O2	• HNG	Reduce ROS Promote CMA	–	(17)
**Humanin-JNK/P38MAPK**	• murine osteoblastic cell line	• H_2_O_2_	• HNGF6A	• Protect osteoblasts from apoptosis and osteoblast phenotype inhibition	–	(60)
	• cortical neurons	• NMDA	• humanin	Reduce ROS Reduce intracellular calcium	• Protect the nervous system	(37)
**Humanin-AMPK**	• HAECs	• FFA	• humanin	• Reduce ROS and NOX2	• Protection of cardiovascular system	(61)
	• Human primary hepatocytes	• Sodium palmitate	• humanin	Reduce ROS Improve glucose and lipid metabolism	Inhibition of lipid accumulation Improve NAFLD	(35)
	Bone marrow- derived macrophage	• RANKL	• humanin	Reduce ROS Inhibit inflammation	• Inhibit bone disorders	(36)
**Humanin-PI3K/AKT-JAK2/STAT3**	• SH-SY5Y neuroblastoma cells	• OGD/R	• HNG	Increase antioxidant enzyme Reduce ROS	• Protect the nervous system	(62)

#### HN Regulates the Expression of the Keap1/Nrf2 Signaling Pathway Through Mitochondria-Nuclear Retrograde Signal Transduction

**Figure 2:**
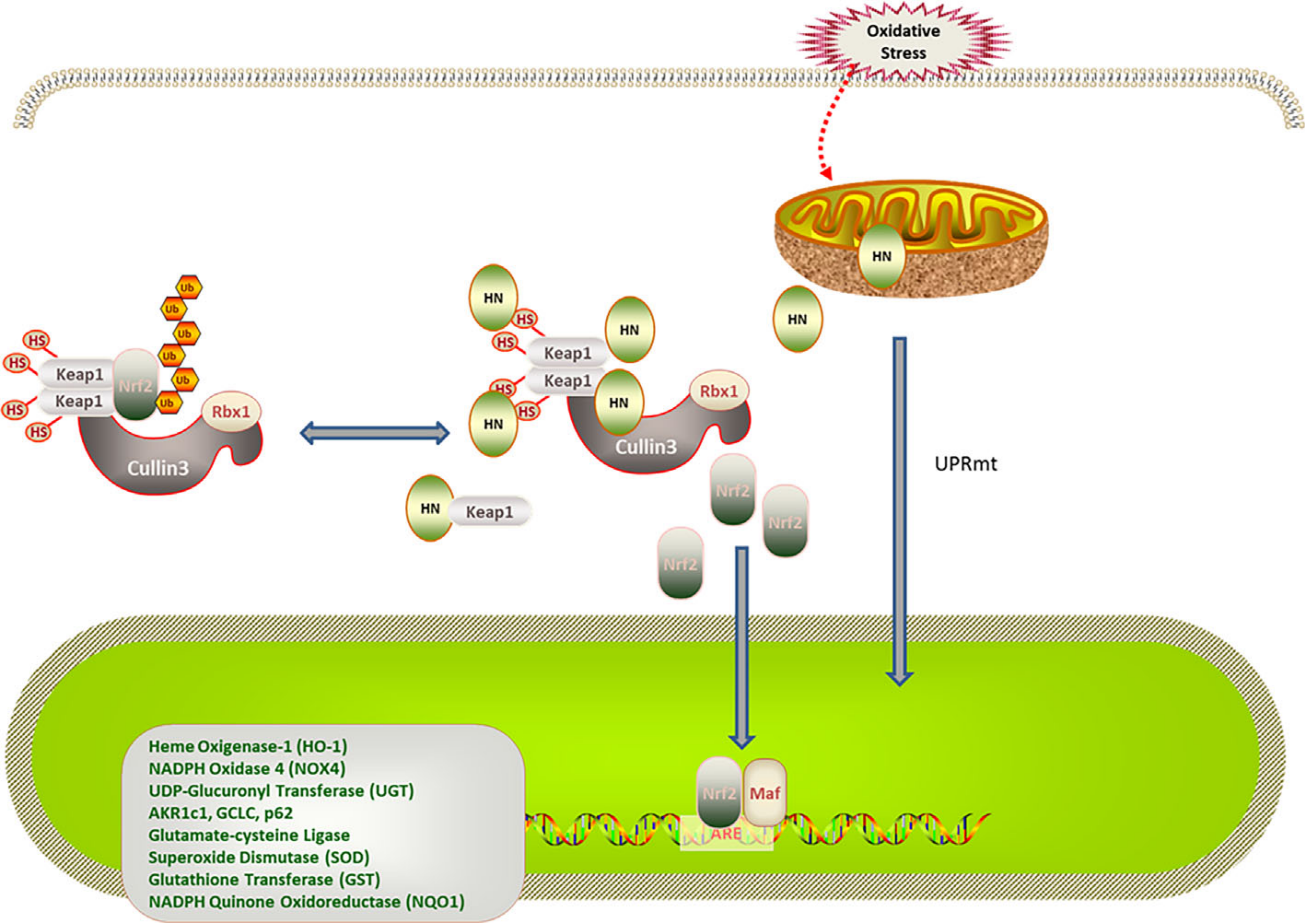
Regulation of HN on Keap1/Nrf2 signaling pathway under oxidative stress. HN promotes Keap1 degradation and release of Nrf2. Under stress conditions, HN inhibits the expression of Keap1, promotes the dissociation of Nrf2 and Keap1, activates the expression of antioxidant genes (SOD, CAD, HO-1, NQO1), and protects mitochondria from oxidative stress damage. Stress also triggers UPRmt, promotes the expression of antioxidant genes and the recovery of mitochondrial function, further promoting the formation of HN.

Nrf2 is a redox-sensitive transcription regulator found in various cells. Under physiological conditions, Keap1 promotes the ubiquitination and proteasome degradation of Nrf2. Under oxidative stress, the conformation of Keap1 is changed by cysteine sulfhydryl modification. Moreover, autophagic degradation of Keap1 is promoted by autophagy-related proteins, thereby increasing free Nrf2 levels in the cytoplasm. After being transferred to the nucleus, Nrf2 binds to antioxidant response elements (AREs), enhancing the expression of antioxidant genes (63, 64). Of note, increased age is associated with decreased Nrf2 stability and reduced antioxidant capacity under oxidative stress (65, 66). Additionally, mitochondria can regulate the expression of nuclear genes through the mitochondria-nuclear retrograde signal transduction pathway. In response to oxidative stress, mitochondria adapt by changing protein expression, resulting in the mitochondrial unfolded protein response (UPR^mt^) (67, 68). UPR^mt^ regulates the expression of nuclear antioxidant genes through the mitochondria-nuclear retrograde signal transduction pathway. UPR^mt^ can be triggered by excessive protein misfolding (67), inhibition of mitochondrial transcription and translation (69), impaired electron transport chain activity, and increased ROS levels (70). Under physiological conditions, the N-terminal of activating transcription factor associated with stress 1 (ATFS-1)/activating transcription factor 5 (ATF5), which is a mitochondria-targeting signal, efficiently mediates the import of ATF5 into the mitochondria. In contrast, the ATF5 import efficiency of mitochondria decreases when they are under stress. The C-terminal of ATFS-1/ATF5 acts as nuclear localization signal, enhancing the ATFS-1/ATF5 import into the nucleus, which promotes the expression of antioxidant enzyme genes in the nucleus, the synthesis of mitochondrial polypeptides, and the recovery of mitochondrial function (71, 72). MOTS-c was the first mitochondrial polypeptide found to regulate nuclear gene expression through a retrograde signal transduction pathway (73). A recent study revealed that HN activates the expression of nuclear antioxidant genes through the Keap1/Nrf2 signaling pathway. In this study, patients with polycystic ovary syndrome were investigated, and a rat model of polycystic ovary syndrome was established. Compared with the control group, the expression of superoxide dismutase (SOD), catalase (CAT), heme oxygenase 1 (HO-1), NADPH quinine oxidoreductase 1 (NQO1), and Nrf2 in the serum, ovarian tissue, and a human ovarian cell line increased; the level of ROS and Keap1 decreased in the group that was treated with the HN analog, HNG (58). Moreover, in a mouse AD model, HNG reduced p62 expression, upregulated autophagy-activating kinase 1, restored the function of cathepsin D, and promoted autophagy in mouse hippocampus tissues. Because Keap1 is ubiquitinated in a p62-dependent manner, it is speculated that HN does not degrade Keap1 by promoting autophagy. Whether HN activates Nrf2 by affecting the conformation of Keap1 needs further study (74, 75) (**Figure 2**).

#### HN and Autophagy Signaling Pathways

Autophagy depends on lysosomal catabolism, which is one of the degradation processes for products of oxidative stress. Autophagy is classified into macroautophagy, microautophagy, and CMA (76, 77). In CMA, the cytosolic heat shock cognate chaperone of 70 kD (HSC70) is involved in recognizing substrate proteins containing a pentapeptide motif, forming a substrate–chaperone complex. The complex is then recognized by lysosome-associated membrane protein type 2A (LAMP-2A), contributing to the transformation of single-span LAMP-2A into a multimeric translocation complex (78, 79). HSP90 at the cytosolic side of the lysosomal membrane facilitates the substrate binding, enhancing the stability of LAMP-2A in the transformation from the monomeric to the multimeric form (78, 79). The luminal chaperone, Lys-HSC70, assists the delivery of the substrates into the lysosome after formation of the translocation complex. Interestingly, oxidative stress and hypoxia are the classical stimulators of oxidation-induced CMA activation that removes oxidized proteins to restore cell homeostasis (80). Impaired CMA leads to the accumulation of oxidative products, increasing oxidative stress damage (81, 82). However, CMA function decreases with age, suggesting a negative association between aging and the antioxidative stress ability (83). Thus, improving the CMA function may be a strategy for treating oxidative stress-related diseases in older adults.

Intriguingly, HN is an endogenous activator of CMA in a dose-dependent manner. One study found that HN protected cells from oxidative stress. This study used NIH3T3 mouse fibroblasts, H9C2 cardiomyoblasts, and MN9D dopaminergic neuronal cells. When oxidative stress was induced, Hsc70 recognized the oxidized protein (substrate), transported it to the lysosomal membrane, and bound to LAMP-2A receptor on the lysosomal membrane. Furthermore, endogenous HN, which is located on the cytoplasmic side of the lysosomal membrane, stabilized the binding of the substrate and the lysosome through Hsp90. With the help of lysosomal cavity chaperone (Lys-Hsc70), the substrate was transported to the lysosomal body and the oxidized protein was removed to maintain cell stability. Thereby, the cell damage caused by oxidative stress was reduced. Moreover, exogenous HNG enhanced the Hsp90-mediated binding of the substrate to the lysosome, upregulated CMA, and reduced oxidative stress damage (17).

**Figure 3:**
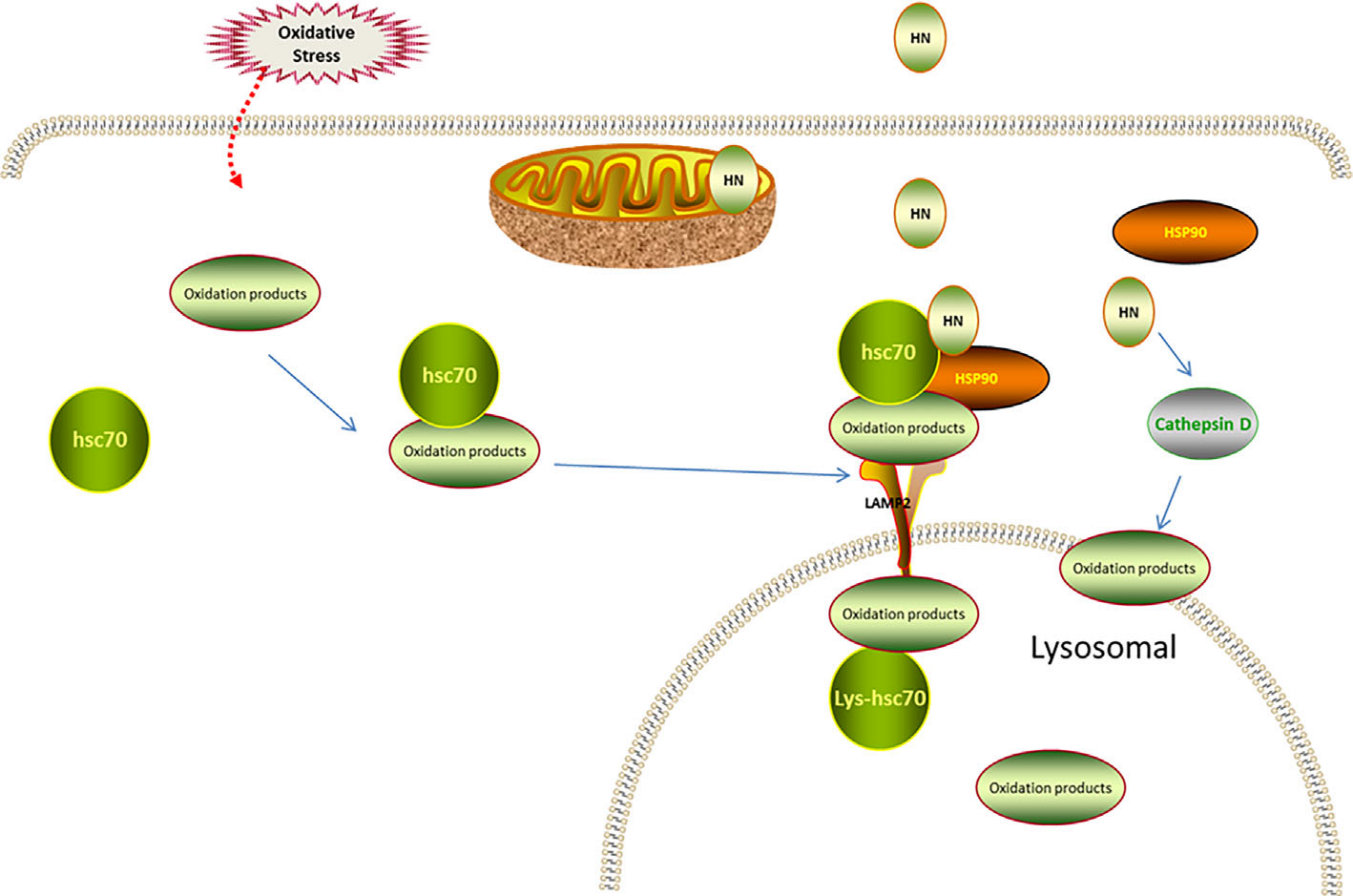
Regulation of HN on CMA under oxidative stress. Under oxidative stress, Hsc70 recognizes the oxidized protein (substrate), transports it to lysosomal membrane, and binds to LAMP-2 receptor on lysosomal membrane. The endogenous HN located in lysosomal membrane cytoplasmic assay stabilizes the binding of substrate and lysosome through Hsp90, and transports the substrate to lysosomal body with the assistance of Lys-Hsc70 to remove oxidized protein. Exogenous supplement of HNG enhances the binding of substrate and lysosome mediated by Hsp90, up-regulates CMA and further reduces oxidative stress damage. HN restores the activity of cathepsin D through FPRL1, promotes autophagy and reduces the production of ROS.

Cathepsin D is also implicated in the HN involvement in autophagic degradation. Cathepsin D, as an intracellular lysosomal restriction inhibitor, is an aspartic protease and cysteine cathepsin. Gly-14 HN restores the activity of cathepsin D through FPRL1, promotes the autophagic degradation of oxidized low-density lipoprotein (ox-LDL) in endothelial cells, reduces the ox-LDL-induced oxidative stress injury of endothelial cells, and decreases the lipid and cholesterol accumulation in endothelial cells (59) (**Figure 3**).

#### HN and the JNK/p38 MAPK Signaling Pathways

**Figure 4:**
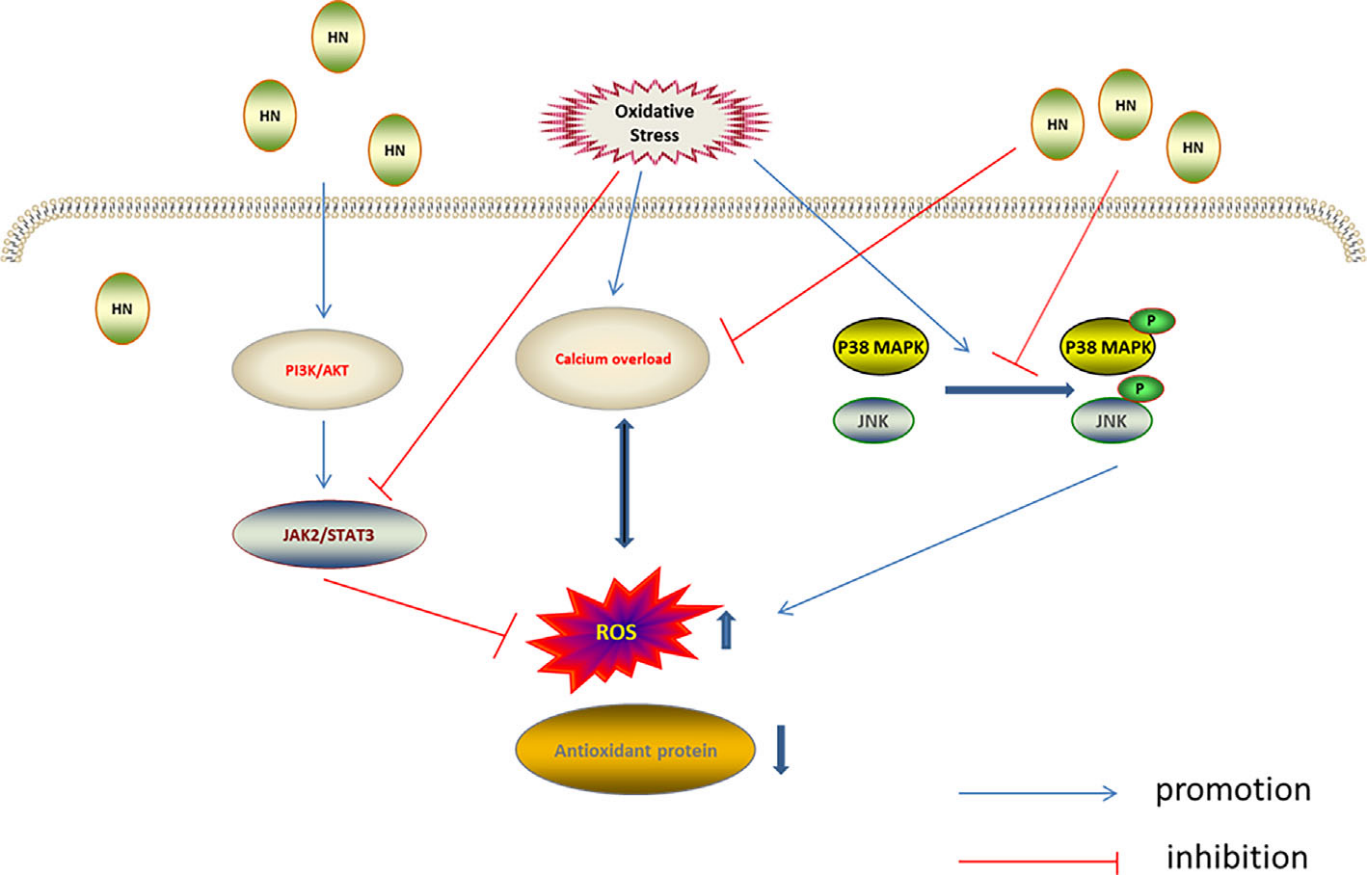
Regulation of HN on MAPKs, PI3K/AKT, and JAK2/STAT3 under oxidative stress. HN inhibits intracellular Ca2^+^ influx, inhibits ROS formation, activates MAPKs inhibitors, inhibits the activation of JNK and p38 MAPK, and reduces oxidative stress damage; HN activates the PI3K/Akt signaling pathway, which further activates the downstream JAK2/STAT3 signaling pathway, promotes the expression of antioxidant protein, reduces the level of ROS, and reduces the oxidative stress damage.

Mitogen-activated protein kinases (MAPKs), including ERK1/2, JNK, and p38 MAPK, are evolutionarily conserved enzymes that connect cell surface receptors and intracellular regulatory targets (84, 85). MAPK inhibition protects cells from oxidative stress (86). Previous studies have found that HN protected neurons by inhibiting JNK and p38 MAPK (87–89). Furthermore, H_2_O_2_ was used to establish an oxidative stress damage model in the murine osteoblast cell line, MC3T3-E1; in this model the HN analog, HNGF6A, decreased ROS production and cell damage caused by oxidative stress by inhibiting JNK and p38 MAPK phosphorylation (60). In another study using the N-methyl-D-aspartate (NMDA)-mediated excitotoxicity model of cortical neurons *in vitro*, HN reduced the release of lactate dehydrogenase, reduced the level of intracellular calcium, inhibited the activation of JNK and p38 MAPK, reduced ROS production by 45.7%, and reduced the oxidative stress injury. The antioxidant mechanism of HN depended on the level of intracellular calcium. Calcium overload leads to increased ROS production, which aggravates the calcium overload. HN weakens the intracellular Ca^2+^ influx, inhibiting the calcium overload and promoting cell survival, indicating that reducing intracellular calcium levels is also required for the HN regulation of JNK and p38 MAPK (37) (**Figure 4**).

#### HN and the AMPK Signaling Pathway

**Figure 5:**
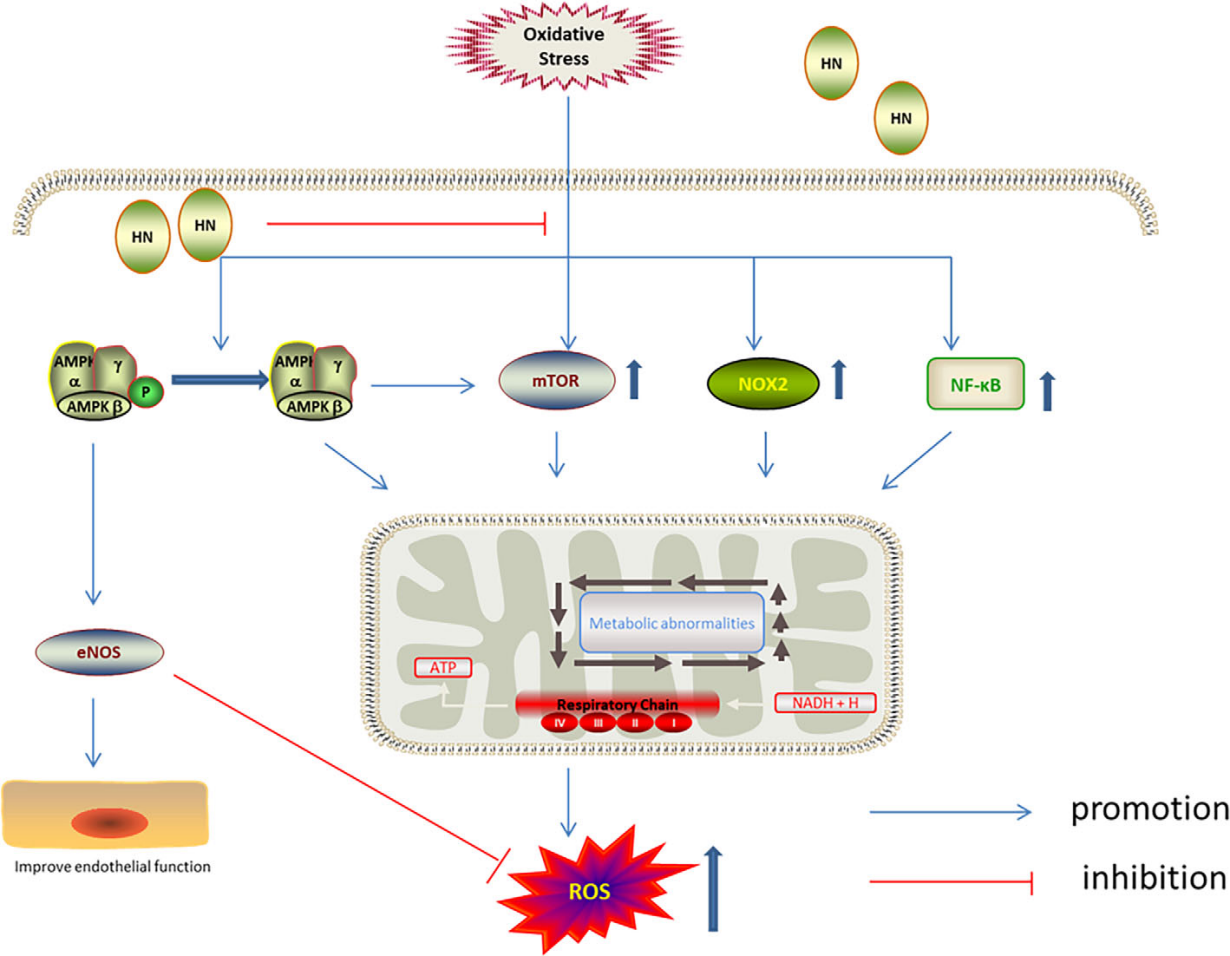
Regulation of HN on AMPK/mTOR under oxidative stress. HN up-regulates the phosphorylation of AMPK, activates eNOS, inhibits mTOR, NOX2 and NF-κB, to reduce the production of ROS, improve endothelial function.

AMPK is involved in the antioxidation effect of HN. AMPK is one of the cell energy sensors in eukaryotic organisms (90); decreased ATP levels activate AMPK, upregulating energy metabolism (90, 91). AMPK keeps cellular metabolic homeostasis by regulating mitochondrial ROS production (92). Interestingly, mitochondria-derived ROS activate AMPK indirectly (93). Compared with wild type mice, increased superoxide and mitochondrial superoxide levels were observed in the aorta of *AMPK*-knockout mice (94). Receptor activator of NF-*κ*B ligand (RANKL) induced differentiation of mouse bone marrow cells, leading to decreased AMPK phosphorylation and increased ROS levels. It has been shown that HN increased AMPK phosphorylation and inhibited the NF-*κ*B pathway, decreasing ROS production and enhancing cell activity (36). HN also inhibited oxidative stress damage in aortic endothelial cells induced by high free fatty acids by activating AMPK. Furthermore, HN reduced the expression of NADPH oxidase 2 (NOX2) and ROS production, inhibited the activation of the inflammatory body, Nod‐like receptor family protein 3 (NLRP3), and thereby protected endothelial cells from oxidative stress damage. NOX2 is the enzyme dedicated to ROS production. It has been demonstrated that HN inhibited ROS production by inhibiting NOX2 (61). AMPK and mTOR play a key role in cell energy metabolism and cell survival. mTOR is a highly conserved serine/threonine kinase, which is involved in regulating cell survival and cell metabolism. mTOR has been reported to play a pathogenic role in insulin resistance and adipogenesis in several cell types. By increasing AMPK phosphorylation, HN inhibited mTOR and regulatory element binding protein 1 (SREBP1) to improve insulin resistance and reduce ROS production (35) (**Figure 5**).

#### HN and the PI3K/AKT, JAK2/STAT3 Signaling Pathways

The JAK2/STAT3 cascade, which is regulated by PI3K/AKT, plays a key role in cell proliferation, anti-apoptosis, anti-aging, cancer development and migration (33, 95, 96). Under oxidative stress, JAK2/STAT3 activity decreases and ROS production increases, leading to increased mitochondrial membrane permeability and cell apoptosis (97). Previous studies have found that HN reactivates the JAK2/STAT3 signaling pathway through the PI3K/Akt pathway, which plays a neuroprotective role (38, 98). Recently, HN has also been shown to reactivate JAK2/STAT3 through the PI3K/Akt signaling pathway to reduce oxidative stress. Furthermore, the neuroblastoma cell line, SH-SY5Y, was used to establish an oxygen/glucose deprivation/reoxygenation (OGD/R) model *in vitro*. In this model, the JAK2/STAT3 signaling pathway was inhibited, the intracellular malondialdehyde (MDA) level increased, and the SOD level decreased. After HNG intervention, the JAK2/STAT3 signaling pathway was activated, the SOD level increased, and the MDA level decreased. Application of HNG + PI3K/Akt inhibitor decreased the levels of JAK2 and STAT3, indicating that the PI3K/Akt inhibitor completely counteracted the activation of the JAK2/STAT3 signaling pathway by HN and suppressed the protective effect of HN (62) (**Figure 4**).

## HN Regulation of the Redox System in ACVD

**Figure 6:**
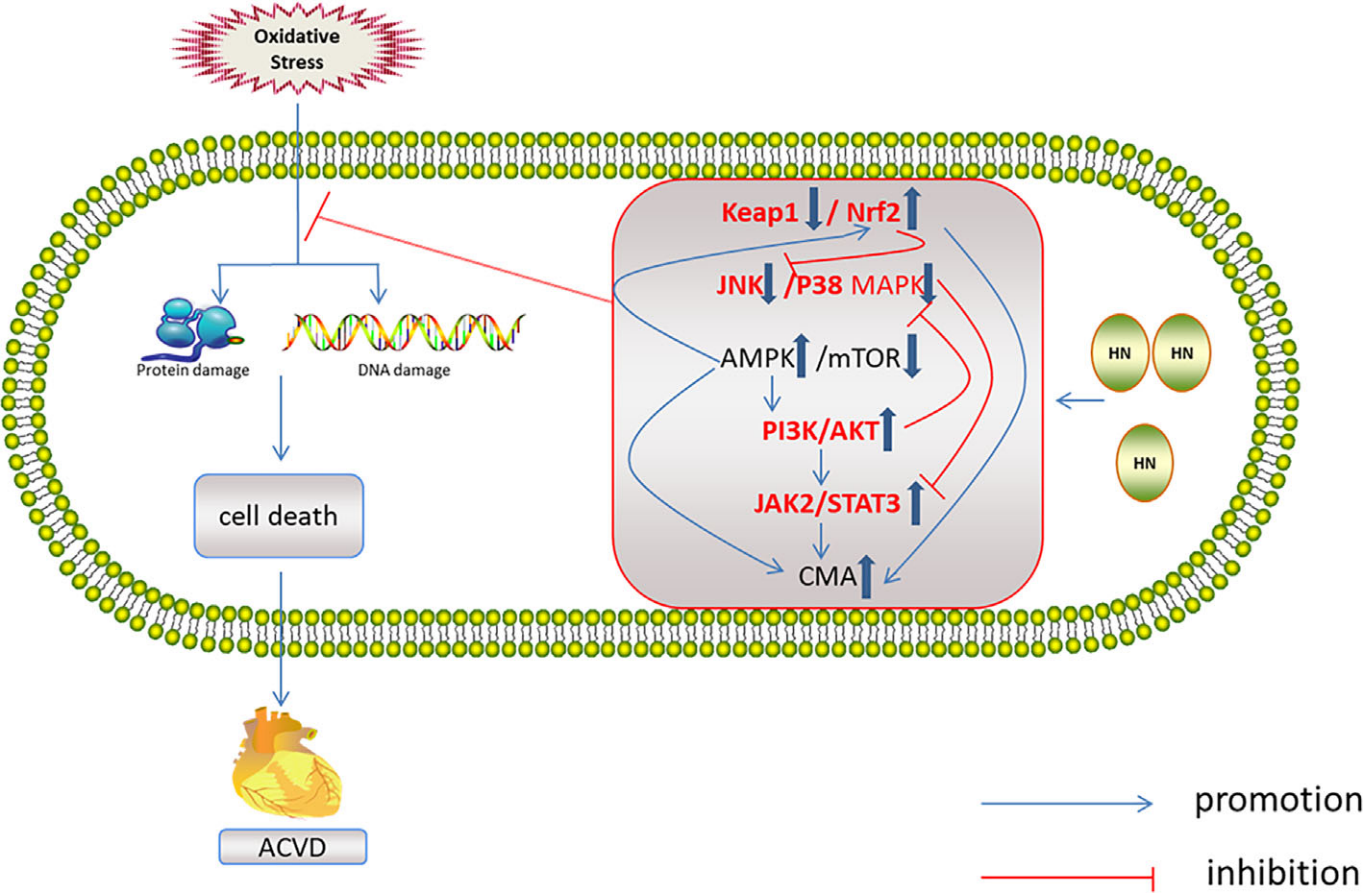
Regulation of HN on redox system in ACVD. HN activates AMPK and PI3K/AKT, JAK2/STAT3 signaling pathways, induces CMA, promotes Nrf2 release, and inhibits JNK/p38MAPK pathway, inhibits oxidative stress damage and ACVD (inside the box: the red font represents the future research direction, the blue thick arrow represents the influence of HN on the five signal pathways, and other signs form a signal network).

HN has protective effects against a variety of cardiovascular diseases, including atherosclerosis (99, 100), acute myocardial infarction, myocardial ischemia–reperfusion injury (45, 99, 101), and myocardium aging (10, 39, 98). The mechanisms of these protective effects all involve oxidative stress (**Figure 6**).

### Arteriosclerosis

Atherosclerosis is an age-related disease. HN and its potent analogs have beneficial effects against age-related diseases (23). Endothelial dysfunction contributes to atherosclerosis (102). Importantly, HN improves endothelial dysfunction through antioxidation, because (1) HN inhibits NOX2, thereby decreasing mitochondrial ROS production; (2) NLRP3 inflammasome activated by mitochondrial ROS leads to endothelial injury; however, HN inhibits the activation of NLRP3 inflammasome by activating AMPK (61).

Hypercholesterolemia is involved in atherosclerosis because ox-LDL infiltrates the subendothelium to form atherosclerotic plaques after endothelial cell injury (103). However, HN prevents the progression of atherosclerotic plaques in hypercholesterolemic mice with apolipoprotein E (APOE) deficiency by reducing the level of nitrotyrosine (NT) and increasing the expression of endothelial nitric oxide synthase (eNOS), which are involved in oxidative stress (14). Ox-LDL is formed by ROS-related oxidation of LDL, ultimately promoting the formation and progression of atherosclerotic plaques by increasing lipid and cholesterol accumulation. Ox-LDL increases the expression of p62 and LC3-II and inhibits the function of cathepsin D activity. HN inhibits the ox-LDL-induced lipid and cholesterol accumulation by decreasing the LC3-II and p62 levels and restoring the ox-LDL-induced cathepsin D functional impairment, thereby reducing the formation of atherosclerotic plaques (45, 104). Lectin-like oxidized low-density lipoprotein-1 (LOX-1) is the main receptor involved in absorption of ox-LDL by endothelial cells. LOX-1 mediates the binding, internalization, and proteolytic degradation of ox-LDL by endothelial cells (105, 106). The HNG-induced decrease in LOX-1 protein expression also contributes to the inhibition of the formation and progression of atherosclerotic plaques (15).

High glucose levels are also implicated in atherosclerosis, leading to endothelial dysfunction. High glucose increases ROS production and the expression of pro-inflammatory factors (tumor necrosis factor-α and IL-1β), further promoting endothelial cells to produce vascular cell adhesion molecule-1 (VCAM-1) and E-selectin. VCAM-1 and E-selectin mediate the adhesion of circulating leukocytes to the endothelium, leading to atherosclerosis development. Kruppel-like factor 2 (KLF2) is involved in endothelial dysfunction induced by high glucose. HN upregulates the *KLF2* gene expression, inhibiting monocyte adhesion to endothelial cells (107).

### Coronary Heart Disease and Heart Failure

Oxidative stress is also involved in the pathogenesis of acute myocardial infarction and ischemia–reperfusion injury. HN protects cardiomyocytes from apoptosis through the antioxidation pathway, reducing the myocardial infarction size and improving cardiac function (108). HN also reduces the necrosis area of myocardial infarction and improves the cardiac function after myocardial infarction by reducing ROS production, which protects the function of myocardial mitochondria (18, 19). HN has been shown to protect isolated myocardial mitochondria from H_2_O_2_-induced oxidative stress. HN increased the levels of GSH, GPX, and SOD, reversing myocardial ischemia–reperfusion injury (12, 20). HNG upregulated the Akt/glycogen synthase kinase-3*β* pathway and inhibited myocardial fibrosis in aged mice (39). It has been found that Nrf2 and Keap1 are necessary for increasing the expression of SOD, CAT, GPX, and GSH. HN may promote the activation of Nrf2 by inhibiting the expression of Keap1 during myocardial infarction (58). In addition, the population study found that compared with normal people, the level of humanin in patients with coronary heart disease decreased and the level of lactic acid increased, suggesting that the protective effect of humanin on cardiovascular system is through antioxidant effect (100). Humanin is positively correlated with coronary artery endothelial function, which may be a target for the treatment of coronary heart disease in the future (99).

Heart failure is the most common complication of myocardial infarction. HN has been shown to decrease the incidence rate of heart failure by inhibiting myocardial hypertrophy (108). Endonuclease G deficiency induces cardiomyocyte hypertrophy by increasing ROS production. Intriguingly, HN has been demonstrated to inhibit cardiomyocyte hypertrophy induced by endonuclease G deficiency (109).

## Relationship Between Redox Signaling Pathways and ACVD

HN reduces oxidative stress through the five above mentioned signaling pathways. These signaling pathways interact with each other and form a network, which is related to ACVD (**Figure 6**). One study has found that Nrf2 deficiency led to aging of human aortic endothelium and mouse aortic endothelium. The aging process was related to autophagy damage. Upregulation of Nrf2 by inhibiting Keap1 activated autophagy and inhibited aging (110). Furthermore, Keap1 upregulation and of Nrf2 inhibition led to oxidative stress damage and aging of vascular smooth muscle cells (111). In a D-galactose-induced mouse aging model, Keap1 expression increased, Nrf2 expression decreased, and ROS production increased (112). Nrf2 inhibits JNK phosphorylation, stabilizes mitochondrial function integrity, and reduces oxidative stress damage (113). In the aging heart, the activation of AMPK and autophagy is impaired. Activation of AMPK induces autophagy, inhibits cardiomyocyte aging, and protects aging myocardium from oxidative stress (114, 115). AMPK activates autophagy by inhibiting mTOR or phosphorylated UKL1 (116), and it activates Nrf2 and protects the myocardium from oxidative stress induced by high glucose (117). AMPK also reduces oxidative stress injury by activating AKT2/Nrf2 (118). Activation of PI3K/AKT by AMPK protects the myocardium from ischemia–reperfusion (I–R) injury (119). Therefore, AMPK is another target for inhibiting myocardial aging (114). Activation of the JAK2/STAT3 signaling pathway inhibits ventricular remodeling after myocardial infarction (120, 121), and it protects the aging heart from I–R injury (122). The JAK2/STAT3 signaling pathway also inhibits cardiomyocyte apoptosis by activating autophagy (123). JNK is the upstream regulator of JAK2/STAT3. Inhibition of JNK activates JAK2/STAT3 and protects the myocardium from oxidative stress induced by high free fatty acids (124). Upregulation of p38 MAPK/JNK phosphorylation promoted NF-*κ*B translocation to the nucleus, induced aging, and aggravated myocardial injury (125). Inhibition of p38 MAPK and JNK phosphorylation protected the heart from oxidative stress injury in aged rats (126). PI3K/Akt inhibits MAPK and NF-*κ*B activation, thereby protecting cardiomyocytes from injury (127, 128). PI3K/Akt inhibits mTOR and protects the myocardium from oxidative stress (129). However, there are few studies on the mechanism of HN and cardiac aging. It is necessary to explore whether HN exerts its anti-aging effects on the cardiovascular system through the above signaling pathways’ network.

## Concluding Remarks and Prospect

Herein, we reviewed the signaling pathways associated with the HN effects against oxidative stress, including the Keap1/Nrf2, the autophagy, the JNK/p38 MAPK, the AMPK, the PI3K/Akt, and the JAK2/STAT3 signaling pathways. We then summarized the relationship among HN, the redox signaling pathways, and ACVD, and pointed out the future research direction for HN and ACVD. Finally, HN may be the target for ACVD treatment by reducing oxidative stress.

## Author Contributions

All authors listed have made a substantial, direct, and intellectual contribution to the work and approved it for publication.

## Conflict of Interest

The authors declare that the research was conducted in the absence of any commercial or financial relationships that could be construed as a potential conflict of interest.
